# BioBricks.ai: a versioned data registry for life sciences data assets

**DOI:** 10.3389/frai.2025.1599412

**Published:** 2025-08-13

**Authors:** Yifan Gao, Zakariyya Mughal, Jose A. Jaramillo-Villegas, Marie Corradi, Alexandre Borrel, Ben Lieberman, Suliman Sharif, John Shaffer, Karamarie Fecho, Ajay Chatrath, Alexandra Maertens, Marc A. T. Teunis, Nicole Kleinstreuer, Thomas Hartung, Thomas Luechtefeld

**Affiliations:** ^1^Center for Alternative to Animal Testing, Johns Hopkins University, Baltimore, MD, United States; ^2^Insilica, Bethesda, MD, United States; ^3^Laboratory for Research in Complex Systems, Menlo Park, CA, United States; ^4^Facultad de Ingenierías, Universidad Tecnológica de Pereira, Pereira, Colombia; ^5^HU University of Applied Sciences Utrecht, Innovative Testing in Life Sciences and Chemistry, University of Applied Sciences Utrecht, Utrecht, Netherlands; ^6^Inotiv, Research Triangle Park, NC, United States; ^7^Renaissance Computing Institute, University of North Carolina at Chapel Hill, Chapel Hill, NC, United States; ^8^Copperline Professional Solutions, LLC, Pittsboro, NC, United States; ^9^Department of Neurological Surgery, Washington University in Saint Louis, St. Louis, MO, United States; ^10^NTP Interagency Center for the Evaluation of Alternative Methods, Research Triangle Park, NC, United States; ^11^Department of Biology, University of Konstanz, Konstanz, Germany

**Keywords:** public health data, BioBricks.ai, data integration, machine learning, cheminformatics, bioinformatics

## Abstract

**Introduction:**

Researchers in biomedicine and public health often spend weeks locating, cleansing, and integrating data from disparate sources before analysis can begin. This redundancy slows discovery and leads to inconsistent pipelines.

**Methods:**

We created BioBricks.ai, an open, centralized repository that packages public biological and chemical datasets as modular “bricks.” Each brick is a Data Version Control (DVC) Git repository containing an extract‑transform‑load (ETL) pipeline. A package‑manager–like interface handles installation, dependency resolution, and updates, while data are delivered through a unified backend (https://biobricks.ai).

**Results:**

The current release provides >90 curated datasets spanning genomics, proteomics, cheminformatics, and epidemiology. Bricks can be combined programmatically to build composite resources; benchmark use‑cases show that assembling multi‑dataset analytic cohorts is reduced from days to minutes compared with bespoke scripts.

**Discussion:**

BioBricks.ai accelerates data access, promotes reproducible workflows, and lowers the barrier for integrating heterogeneous public datasets. By treating data as version‑controlled software, the platform encourages community contributions and reduces redundant engineering effort. Continued expansion of brick coverage and automated provenance tracking will further enhance FAIR (Findable, Accessible, Interoperable, Reusable) data practices across the life‑science community.

## 1 Introduction

The integration of artificial intelligence (AI) into toxicology and biochemistry is revolutionizing these fields, enhancing data analysis capabilities and contributing to more efficient and accurate insights. AI methods excel at processing large, diverse datasets, which are increasingly valuable for modern toxicology and biochemistry research (Lin and Chou, 2022; Hartung, 2023). In toxicology, AI-powered predictive tools like Read-Across Structure–Activity Relationships (RASAR) have achieved 87% balanced accuracy across nine Organisation for Economic Co-operation and Development (OECD) tests and 190,000 chemicals, surpassing traditional methods in predicting chemical toxicity (Luechtefeld et al., 2018). Large language models are making a growing impact on chemistry, with the capacity to predict chemical properties, evaluate synthesis pathways, and generate compounds optimized to reduce toxicity (Ramos et al., 2024). These models require and benefit from large amounts of data, but many of the same datasets used for these assets are laboriously collected repeatedly from different research groups (Fabian et al., 2020; Lee et al., 2020; Yüksel et al., 2023). The power of AI in fields such as toxicology and biochemistry depends heavily on the quality, quantity, and accessibility of data. Standardized data access is essential for integrating diverse data types, ensuring reproducibility, and facilitating the training and validation of AI models. Standardization supports cross-disciplinary research, regulatory compliance, and efficiency by reducing the time researchers spend on data preparation. The lack of large, high-quality training datasets is a critical barrier to the broader application of AI models in fields such as public health (Jain et al., 2024). The demand for data often surpasses the pace at which new datasets are generated and made available, highlighting the need for better data collection, management, and sharing practices (Alzubaidi et al., 2023).

There are many independent databases for public health. The European Bioinformatics Institute’s identifiers.org, a registry for biomedical datasets, lists 838 such distinct data sources. This is by no means an exhaustive list, but illustrates the diverse landscape of available public health information (NIH LIBRARY, n.d.; Centers for Disease Control and Prevention, 2021). A survey of data scientists performed in 2022 reported that about 38% of developer effort is spent on accessing and cleaning data, rather than modeling and analyzing it (ANACONDA, 2022, Proto-OKN, n.d.), thus wasting valuable resources and working hours.

BioBricks solves the problem by providing a package manager for data. It provides a standardized format that works well with developer tools and allows users to have a single location to search for and install data assets. By streamlining data management and distribution, BioBricks.ai has the potential to accelerate the pace of progress in the life sciences. It reduces barriers to data access, collaboration, and distribution, allowing researchers to focus on analysis and innovation rather than data preparation and management. Herein, we provide a detailed overview of BioBricks and describe several application use cases.

## 2 Methods

### 2.1 BioBricks.ai overview

BioBricks.ai aims to simplify the provisioning of this training and evaluation data. With a few lines of code, datasets can be loaded into a computation environment. BioBricks.ai provides a public, centralized Data Version Control (DVC)^1^ data registry for public health data assets (Data Version Control, n.d.-a; Data Version Control, n.d.-b). While built on DVC for data science projects, BioBricks.ai enhances this foundation with a specialized command-line tool and web portal focused on installing and managing data dependencies in a manner akin to package managers like the Comprehensive R Archive Network (CRAN), Bioconductor and PyPI (Bommarito and Bommarito, 2019; Dong et al., 2021).

BioBricks.ai manages data assets organized into ‘brick’s. Each brick is a git repository adhering to a standardized protocol outlined in the BioBricks.ai template repository.^2^ Bricks can be created with or without dependencies on other bricks. For independent bricks, which often represent primary data sources, BioBricks.ai’s policy is to replicate the original data without modifications, ensuring data integrity and fidelity, with full attribution and citation. Examples include the HUGO Gene Nomenclature Committee brick^3^ and the ClinVar brick,^4^ a database of clinical variants and their relationship to human health, (Bruford et al., 2007; Landrum et al., 2016).

Bricks can also be built with dependencies on other bricks, like these primary sources, allowing for more complex data structures that might restructure data, combine multiple sources, or generate derived products like machine learning models. This flexible structure enables BioBricks.ai to maintain a hierarchy of data resources, from raw datasets to sophisticated, integrated products. A prime example is the ChemHarmony brick, which combines and simplifies data from over fifteen chemical-safety**–**related databases into a single, unified schema using unique, curated chemical identifiers. By providing standardized access to consistent versions of datasets, BioBricks.ai significantly reduces data acquisition time, facilitates collaboration among researchers, and simplifies the process of building downstream assets that depend on multiple upstream data sources.

With a straightforward installation process, the BioBricks.ai tool offers a unified interface to discover and utilize numerous data sources. Instead of navigating multiple databases, APIs, packages, or specialized data tools for each new source, researchers only need to learn one straightforward system. The accompanying web application, https://biobricks.ai, enables tracking of asset usage, potentially facilitating future features like bandwidth cost allocation and enhanced tooling around constructed data sources.

BioBricks.ai can be used to quickly install ‘bricks’, which are git repositories with code for building databases (or other data assets). Getting set up involves installing the command line tool, configuring the tool, and then installing bricks (see Code 1):

**CODE 1:**
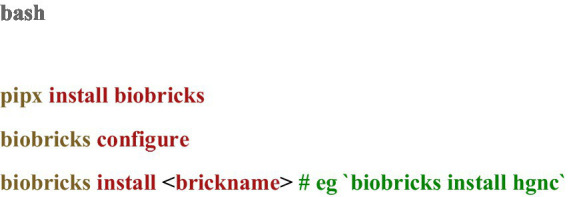
Configuring BioBricks and installing a brick can be done in 3 steps.

BioBricks.ai recommends using pipx to install the command line tool in an isolated environment. Pipx is a command-line utility that enables users to install Python packages into isolated environments. By using pipx to install the BioBricks command line tool, users can run commands such as biobricks install without the need to manage dependency conflicts with other Python environments. Alternatively, users can install BioBricks.ai using pip install biobricks if preferred. The tool is designed to be lightweight, with minimal dependencies, ensuring a simple and efficient installation process. Importantly, while DVC is used in the development of bricks, it is not required for end-users of the BioBricks.ai tool, further streamlining the user experience for researchers who need only to access and utilize the data.

Researchers can find all available bricks developed by the BioBricks.ai team on GitHub at https://github.com/biobricks-ai and on the official website at https://biobricks.ai. To use the tool, users create an account at BioBricks.ai, which provides them with a token that enables asset downloads. Although brick downloads are free today, each user must register for a token so that the platform can (i) record anonymized usage statistics, (ii) enforce fair-use limits that deter scraping/abuse, and (iii) keep open the option of cost-recovery (pay-as-you-go for multi-terabyte transfers) should future bandwidth bills demand it. The login layer is therefore a governance safeguard, not a paywall, ensuring BioBricks.ai remains sustainable while remaining free for the community. Several example bricks are shown in Table 1. Bricks are categorized based on the data they contain: Chemical Informatics, Cancer Research, Genomics & Genetics, Proteomics, Pharmacology and Drug Discovery, Toxicology and Environmental Science, Medical and Clinical Sciences, Ontology and Terminology, and Systems Biology and Pathways. A knowledge graph visualization of each brick and their associated category is shown below in.

**Table 1:** Examples of databases in BioBricks.ai.

Repository	Description
ice	Integrated Chemical Environment–High quality in vitro and *in vivo* toxicology data.
biogrid	Data from BioGRID.
ctgov	Data from ClinicalTrials.gov.
mirbase	Data from miRBase.
skinsensdb	Skin sensitization database.
ctdbase	Data from Comparative Toxicogenomics Database.
tox21	Tox21 quantitative high throughput screening (qHTS) 10 K library data.
targetscan	Data from TargetScan.
USPTO_ChemReaction	Data from USPTO Chemical Reaction Database.
moleculenet	Molecular datasets for machine learning.
pubchem	PubChem data.
ToxValDB	Toxicity endpoint data.
dbgap	Genotype–phenotype interaction data.
zinc	ZINC purchasable compound database.
toxcast	EPA in vitro toxicity data.
pdb	Protein Data Bank 3D structure data.
geneontology	Gene Ontology knowledgebase.
cpdat	Consumer Product Data.
cpcat	Chemical Product Categories.
chembl	Bioactive molecule data.

Documentation for building, installing, and configuring BioBricks.ai is available at https://docs.biobricks.ai. In the following sections, we provide some details on how BioBricks.ai functions, but we refer active users to the documentation for complete details.

### 2.2 Architecture and design considerations

BioBricks.ai was designed to address key challenges in life-science data management, such as handling very large datasets, preserving version history for reproducibility, and simplifying data integration from diverse sources. The system architecture centers on a local “brick library” directory (specified by the user) within which each installed dataset (or “brick”) is stored in a structured manner. To avoid duplicating large files, BioBricks.ai employs a content-addressable storage strategy: all data files are identified by an MD5 hash and stored in a central cache subdirectory. This hashing mechanism ensures that each unique file is downloaded only once, even if required by multiple bricks, thus minimizing disk usage and download time. Each brick itself is implemented as a Git repository conforming to a standard template, enabling version control of both the dataset contents and its build pipeline. The repository’s path in the library encodes the brick’s source organization, name, and commit hash (version)—for example, a path ./biobricks-ai/chemharmony/4f060 corresponds to version 4f060 of the “chemharmony” brick under the biobricks-ai organization. This hierarchical structure allows multiple versions of a dataset to coexist and ensures that every brick installation is precisely reproducible.

A key architectural decision was to build BioBricks.ai on top of Data Version Control (DVC) and Git. Each brick repository includes a DVC pipeline that automates the extract-transform-load (ETL) steps for constructing the dataset from its original source(s). This approach captures provenance and enables automated rebuilding of bricks when source data are updated. We chose to standardize on a small set of optimized data formats—Parquet tables for structured tabular data, SQLite databases for smaller or indexed data, and HDT for semantic triple stores. These formats were selected for their balance of efficiency, interoperability, and support in common data science tools. For example, Parquet provides compression and partitioning that are advantageous for big data analytics (enabling faster parallel downloads and distributed processing), while SQLite offers a lightweight, self-contained database with robust indexing for fast queries on medium-sized datasets. By narrowing to these formats (without precluding others if needed), the design simplifies downstream use of BioBricks data in AI/ML workflows.

From the outset, we emphasized usability and minimal setup as design goals. BioBricks.ai’s command-line interface (CLI) provides a unified way to search and install data assets in a manner analogous to package managers like CRAN or pip. A user can install a brick with a single command, and behind the scenes the system handles locating the repository (defaulting to the central BioBricks.ai registry on GitHub), cloning it, retrieving the data files from cloud storage, and linking them into the local brick directory. The installation and configuration instructions (originally included in the Methods) have been moved to Appendix A. In place of those technical steps, we now focus on design rationale. For instance, by caching files and linking them to brick directories, and by leveraging DVC’s incremental updates, BioBricks.ai can manage multi-terabyte datasets more practically. We recognize that downloading an entire multi-terabyte asset is impractical for many users; thus, a planned enhancement is to support cloud-based queries and partial dataset retrieval (so that users can work with subsets of data without full downloads—see *Limitations*). Nonetheless, the current architecture lays the groundwork by eliminating redundant transfers and enabling content-based updates.

Another design consideration was supporting complex data dependencies and community contributions. BioBricks.ai allows bricks to declare dependencies on other bricks, meaning one dataset can be built using other bricks as inputs. This modular approach encourages reuse: for example, a derived brick can integrate data from several primary-source bricks without duplicating their content. All such relationships are explicitly recorded (each brick repository contains a .bb/dependencies.txt file listing required bricks by URL or identifier), and the BioBricks CLI automatically ensures that prerequisites are installed and up-to-date when a brick is installed. This hierarchy of bricks was a deliberate architectural choice to reduce redundant effort and to enable community-driven expansion of the registry. A contributor can focus on crafting a new integrated dataset or analysis, while BioBricks.ai takes care of fetching the correct versions of underlying data. Currently, new bricks are added to the official registry via an invitation and review process with the core team to ensure quality and compliance. To further open this process, we are developing a biobricks push workflow that will allow any authenticated user to submit a new brick for review. This submission pipeline will include automated quality assurance (e.g., schema validation, virus scan, license checks), after which approved bricks are merged into the central catalog and become searchable on the platform. By balancing open contribution with curation, the design aims to grow the BioBricks.ai dataset collection in a controlled yet community-driven manner (Technical details on installing and using the BioBricks.ai CLI are provided in Appendix A).

## 3 Data formats

Currently, BioBricks.ai supports three primary data types: Parquet, SQLite, and HDT (Header, Dictionary, Triples). These formats were chosen for their specific advantages in handling different types of data and supporting various use cases: We refer to Parquet and sqlite bricks as ‘tabular-bricks’ and HDT as ‘triple-bricks’. The system can distribute any serializable data format, but these formats are preferred; features specially built on these data types may be implemented in the future.

### 3.1 Parquet

BioBricks.ai supports Parquet for its compression capabilities, which significantly reduce the size of data files. Parquet also supports partitioning such that one large table can be partitioned into many smaller Parquet files. Compression and partitioning are important for network efficiency, as data can be partitioned into smaller files that are faster to download in parallel. Partitioning is also important for distributed computing systems like Spark and Dask, which are often used to process BioBricks.ai assets (Parquet Files, n.d.; Apache Parquet, n.d.).

### 3.2 SQLite

SQLite is used within BioBricks.ai for its robust indexing capabilities and self-contained nature. This simple, serverless database system makes it easy to manage. Its indexing features facilitate quick data retrieval, which is beneficial for operations that require fast access to data (SQLite, n.d.-a; SQLite, n.d.-b). SQLite’s portability and ability to handle complex queries make it an ideal choice for researchers who need to perform detailed data exploration without the overhead of a full database management system.

### 3.3 HDT (Header, Dictionary, Triples)

BioBricks.ai adopts HDT for managing semantic knowledge graphs. HDT optimizes the storage and querying of RDF (Resource Description Framework) datasets by compressing RDF data and organizing it effectively. This structure supports efficient graph operations and accelerates both data loading and complex querying (Fernández et al., 2020; RDF HDT, n.d.). HDT is particularly valuable for projects that involve linked data or require semantic reasoning capabilities.

The Parquet, SQLite, and HDT formats were chosen over others due to their balance of efficiency, flexibility, and widespread support in data science tools and libraries. While BioBricks.ai can distribute any serializable data format, these three formats are preferred for their optimal performance in various data processing scenarios. The system may implement special features built on these data types in the future, further leveraging their unique strengths.

## 4 Results

### 4.1 Capabilities and performance

#### 4.1.1 Registry content

BioBricks.ai has grown into a sizable data resource. As of this writing, the public registry hosts over ninety versioned datasets (“bricks”) encompassing a range of biological and chemical data sources. These bricks include genomic references, chemical toxicology databases, clinical datasets, and other public health data assets contributed by the community. Each brick can be installed on-demand via the CLI, providing researchers with a convenient “one-stop” data access mechanism.

#### 4.1.2 Usage metrics

At present, we do not have usage statistics to report for the platform. BioBricks.ai does not yet track downloads or active usage of specific bricks—it operates as an open tool without built-in analytics. We acknowledge that understanding how the community uses the bricks is important for guiding future development. Therefore, we plan to implement anonymized tracking and user analytics in a future update. This will likely include opt-in metrics such as the number of times a given brick is installed or updated, which can help us identify popular assets and prioritize maintenance or improvements. In this paper, we focus on the features and content of BioBricks.ai, and we state upfront that usage data is currently unavailable. Establishing a feedback loop with user metrics is part of our roadmap to ensure the platform continues to meet researchers’ needs.

### 4.2 Use cases

#### 4.2.1 Create novel data assets with dependencies on other assets

BioBricks.ai is useful for creating new assets that depend on one or more other bricks. The ChemHarmony brick, https://github.com/biobricks-ai/chemharmony, combines many chemical-property data assets into a single, simplified asset primarily created for modeling chemical properties.

**Figure 1:**
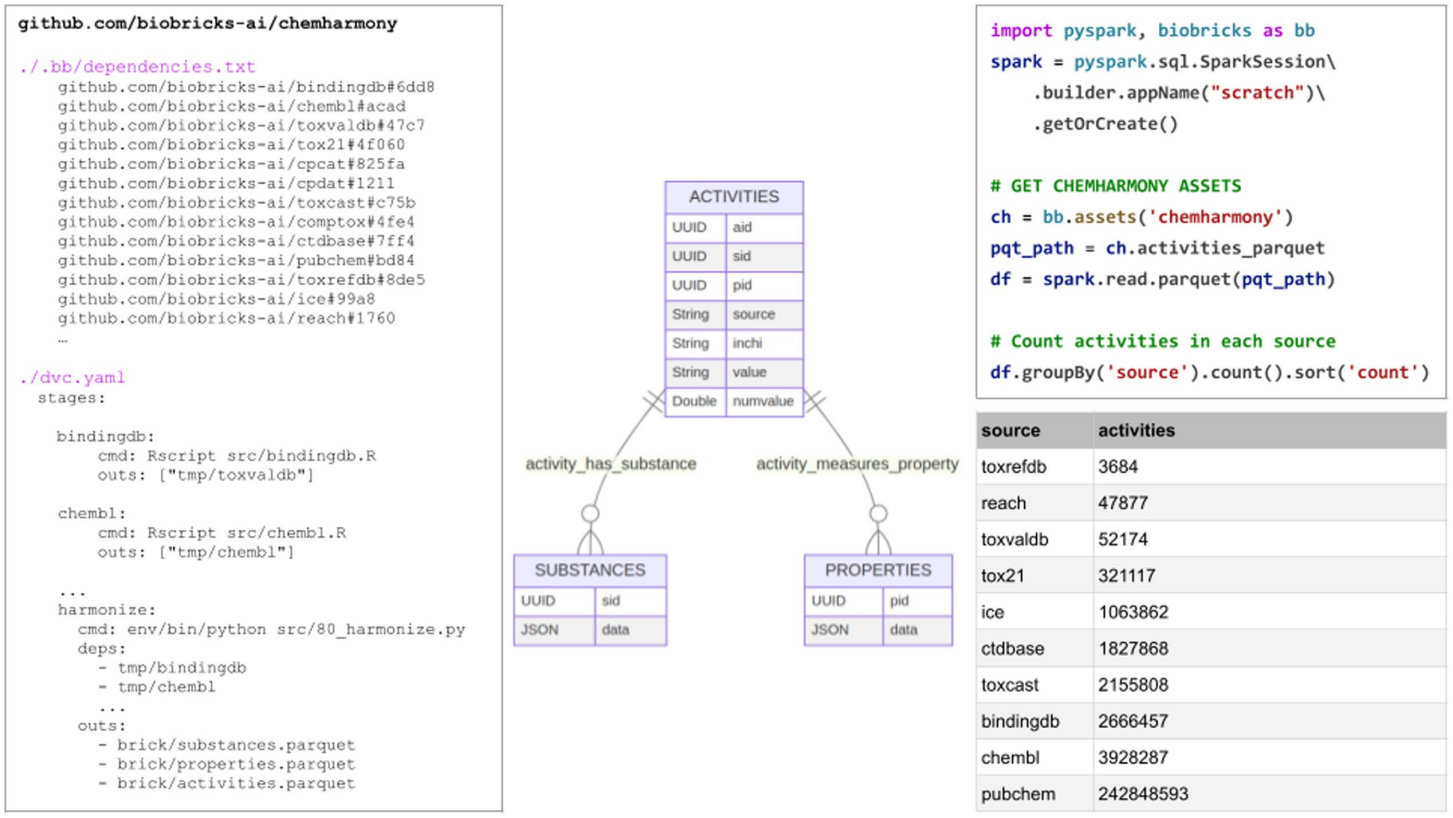
Left–truncated versions of the (1) .bb/dependencies.txt and (2) dvc.yaml file in the ChemHarmony BioBrick. Center, the 3-table schema of ChemHarmony, a simple chemical activities dataset with a substances, properties, and activities table. Right shows how to count activities by source by installing the ChemHarmony brick and using it with Apache Spark with the resulting table in lower right.

The ChemHarmony project is designed to integrate chemical-property values from various databases into a unified system. The database is structured into three main tables: substances, properties, and activities. Each activity links a substance to a property with an assigned value, either binary (indicating positive or negative) or numerical (such as binding affinity or LD50 values), facilitating a quick assessment of chemical characteristics. As shown in Figure 1, the properties table contains the property ID (pid) column and a JSON column containing metadata describing the property. The substances table contains substance ID (sid) and a JSON column describing substance-metadata provided by the substance source. The activities table connects the sid and pid; it also provides structural information such as SMILES and InChi to make it easier to build downstream modeling bricks. The chemharmony code contains scripts to process every source database into the substances, properties, and activities tables thus reducing many complex heterogeneous tabular schemas into one simple schema.

BioBricks.ai supports ChemHarmony by providing the infrastructure and tools necessary to integrate chemical-property data from various sources into a unified database. The databases in ChemHarmony include ChemBL, eChemPortal, ToxValDB, Tox21, CPCat, CPDat, ToxCast, CompTox, CTDbase, PubChem, QSAR Toolbox, BindingDB, ToxRefDB, ICE, and REACH, with several more additions in progress, including RTECS, PubChem-annotations, and Clintox (COMPTOX_Public, n.d.; Gaulton et al., 2017; Zanzi and Wittwehr, 2017; Science inventory, n.d.; Xu et al., 2023; Dionisio et al., 2015; Dionisio et al., 2018; Richard et al., 2016; Davis et al., 2023; Kim et al., 2016; Mombelli and Pandard, 2021; Gilson et al., 2016; Watford et al., 2019; Bell et al., 2017; Sweet et al., 1999; ClinTox, n.d.; Kim et al., 2016). ChemHarmony includes 117 million chemicals and 254 million activities, with 4,026 major properties having over 1,000 activities each, including 246 million activities and 4.1 million chemicals in these major properties.

ChemHarmony has already gone through several revisions, with more to come. The BioBricks system made it easy for a team of people working on this asset to collaborate without worrying about synchronizing data dependencies between developer environments. This also means that releases of the ChemHarmony asset have unambiguous dependencies on upstream databases. This same system can be used to indicate when ChemHarmony needs updating and trigger a rebuild. Figure 1–Left shows a truncated version of the .bb/dependencies.txt ChemHarmony file. This file is built when a user runs biobricks init within a BioBricks.ai repository. It references the git repo and commit hash of each asset that the repository depends on. When a user runs biobricks pull within this repository, all of the bricks they need, but do not currently have, are installed. This allows all ChemHarmony developers to maintain a standardized environment.

When dependencies change, ChemHarmony can be updated easily through manual modification of the .bb/dependencies.txt file or by running biobricks add <brick>, ensuring that all components are up-to-date without disrupting the workflow. All developers work with the same version of the data, thanks to the standardized management of dependencies and data integration provided by BioBricks.ai. This consistency improves collaboration and reduces errors.

#### 4.2.2 BioBricks.ai accelerates the exploitation of existing knowledge

Scientific literature provides a valuable source of information about the relationships between biological and chemical entities, which can, for example, support drug repurposing or the discovery of unforeseen drug adverse events. A significant portion of this literature is gathered in PubMed through publicly available abstracts. However, regular changes to the API, as well as a rate-limiting process, make it challenging to analyze the entirety of the corpus. Downloads are possible in the form of FTP bulk transfers, but these need to be stored in a dedicated platform, which might be disconnected from the analysis environment.

**Figure 2:**
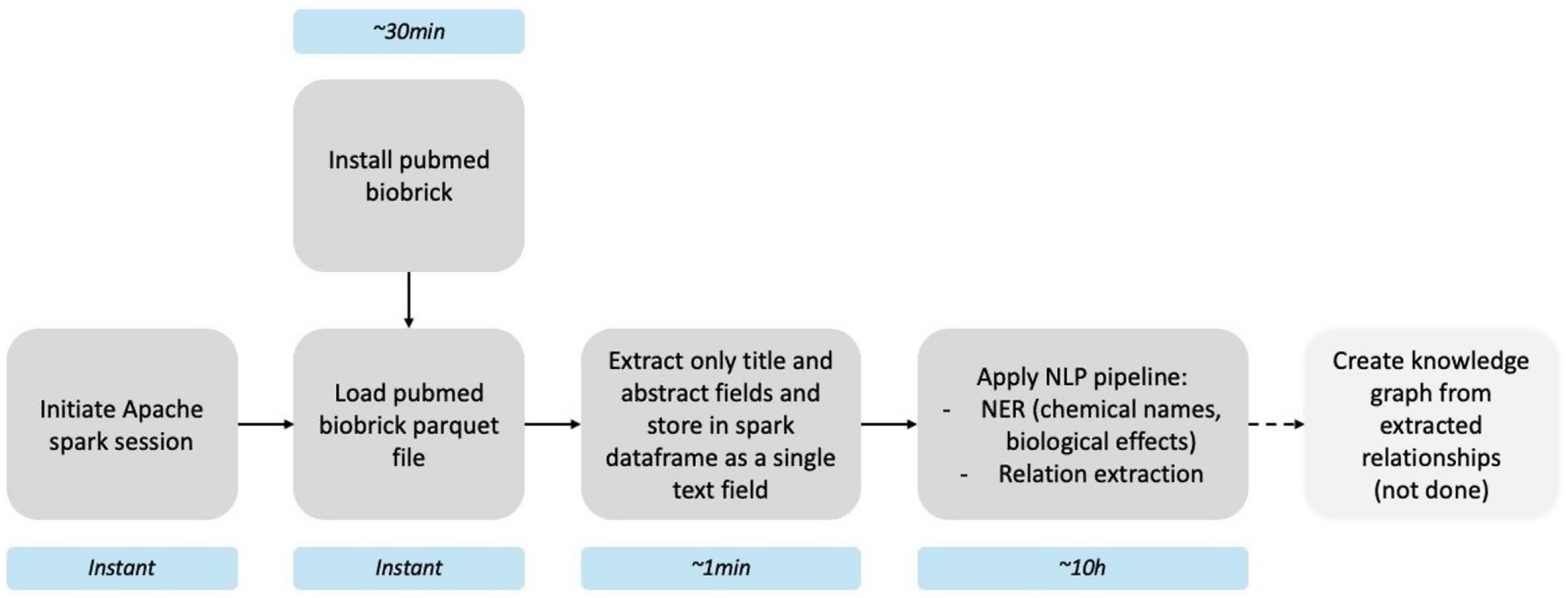
Pipeline for scientific knowledge extraction using the PubMed brick. Light blue boxes show the approximate runtime of each stage.

The PubMed brick (≈ 66 GB) lets us apply a Spark- and spaCy-based NLP workflow to every PubMed abstract to extract chemical entities, adverse effects, and their putative relationships. After installing the brick (~30 min), we initiated an Apache Spark session and loaded the parquet asset (instant). Titles and abstracts were concatenated into a single text column (~1 min) and processed with a custom pipeline—named-entity recognition followed by relation extraction—to append chemical–effect pairs to each record (~10 h). An optional final step, creating a knowledge graph from extracted relationships, is outlined in Figure 2 but was not run in this demo. The complete pipeline script is available at https://github.com/ontox-hu/pubmed-entox; further methodological details are given in Pampel et al. (2023). Because the brick is distributed in an append-only format, future installs retrieve only new data, keeping refresh times minimal.

## 5 Discussion

### 5.1 Comparison with existing technologies

The current landscape of public data asset management in the life sciences is highly fragmented, with numerous independent data sources each using their own ad-hoc distribution mechanisms (APIs, FTP servers, custom formats, bespoke packages, etc.). Data users often find themselves writing redundant extract-transform-load (ETL) code for each source. For example, a researcher studying drug interactions might need to navigate NCBI’s web interface for genomic data, use ChEMBL’s API for chemical information, and parse custom file formats from toxicology databases—each requiring separate scripts. Two common strategies have emerged to address these integration challenges: federated data networks and harmonization (integrated) data assets.

*Federated data approaches* (exemplified by the Semantic Web paradigm) rely on each data provider adopting shared standards and ontologies. In theory, this yields a distributed ecosystem where normalized queries can seamlessly span multiple datasets via technologies like SPARQL. In practice, however, the *coordination overhead* and technical complexity of enforcing a common ontology across diverse groups is a significant barrier. While resource-intensive, federation can succeed in specific domains. Federated discovery tools also exist: the re3data registry indexes over 3,000 research data repositories worldwide (Pampel et al., 2023), and the NIH’s DataMed initiative built an open-source Data Discovery Index that harvests and unifies metadata from dozens of biomedical data repositories (Chen et al., 2018). These efforts greatly improve the *findability* of datasets, but they do not actually unify data formats or eliminate the need for custom processing code—users still must retrieve data from each source and handle idiosyncratic formats.

Harmonization assets involve groups building new assets to simplify and reduce heterogeneity in the life-science data ecosystem. Examples include PharmacoDB (Feizi et al., 2022), Harmonizome (Rouillard et al., 2016), ROBOKOP (Fecho et al., 2021), Hetionet (Himmelstein et al., 2017) and SPOKE (Morris et al., 2023). These sources take upstream data sources and develop code to extract, transform, and load those sources into new data assets. While this approach reduces coordination costs by moving the burden of data transformation to the developer of the new harmonized asset, it also adds complexity to the data ecosystem. Each new asset creates maintenance costs, and the proliferation of such assets often results in redundant efforts.

Table 2 provides a high-level comparison of these approaches (federated semantic-web initiatives, harmonization assets, and the traditional status quo of siloed repositories) versus BioBricks.ai. Traditional data repositories here refer to conventional static sources like journal supplement files, legacy FTP servers, or standalone databanks that offer data dumps with minimal tooling. These typically lack version control, have infrequent or manual updates, and require end-users to perform their own integration. For example, Gene Expression Omnibus (GEO) provides bulk data via FTP, and many journals still distribute datasets as static CSV/ZIP attachments—convenient for sharing, but burdensome for reuse and prone to being out-of-sync. By contrast, BioBricks.ai is designed to enable continuous versioning, standardized access interfaces, and automated pipelines for data retrieval and processing.

**Table 2:** Comparison of features of BioBricks.ai with other approaches to data distribution in life sciences.

Feature	BioBricks.ai	Federated data approaches (semantic web)	Harmonization assets	Traditional data repositories
Data integration	Supports multiple data types and sources	When standards are adopted	Within specific domains	Often siloed
Standardization	Standardized access, flexible data formats	Requires coordination on ontologies	Within asset scope	Varies widely
Ease of use	Package manager-like system	Requires specialized knowledge	Depends on asset	Often requires manual navigation
Reproducibility	Version control built-in	Depends on implementation	Within asset scope	Often lacks versioning
Scalability	Designed for large datasets	Depends on infrastructure	Asset-dependent	Often limited by original design
Community contribution	Open-source model	Depends on governance	Often centrally managed	Usually closed systems
Data update frequency	(Not yet implemented) Can be real-time in future	Depends on participants	Often periodic releases	Varies widely
Interoperability	Common access method for diverse data	When standards are adopted	Within asset scope	Often requires custom integration
Learning curve	New system, but designed for ease of use	Requires understanding of complex standards	Asset-specific knowledge needed	Varies - Often high for each new source
Cost efficiency	Reduces redundant work	High initial investment	Reduces some redundancy	Often leads to redundant work
Flexibility for new data sources	Can easily add new ‘bricks’	Requires adherence to existing standards	Often limited to predefined scope	Can add, but often in isolation
Support for AI/ML applications	Designed with AI/ML needs in mind	Depends on data quality and format	Often designed for specific AI/ML tasks	Often requires significant preprocessing

It provides a side-by-side feature comparison: cells are color-coded with Green for strong support, Yellow for moderate/partial support or issues, and Red for little to no support or significant limitations in that area. These general ratings may vary for specific implementations or use cases.

Importantly, neither federated networks nor one-off harmonized databases adequately solve the *“long tail”* problem of data integration: when researchers want to use a niche or new data source that is not yet supported, they must fall back on writing custom pipelines. BioBricks.ai aims to fill this gap by providing a package manager-like data layer that makes adding new data sources easier and promotes reuse of ETL efforts. BioBricks.ai is essentially a collection of Git repositories (each called a “brick”) that contain the code to download, parse, and transform a given data source into a standard format. This model dramatically *reduces coordination costs*—data publishers or community contributors can encapsulate their source in a brick without needing all providers to agree on global standards. Once a brick is created, its ETL pipeline can be reused by any number of downstream users. BioBricks thus turns what is typically days of ad-hoc data wrangling into minutes of installation. For example, one contributor spent roughly 1 week developing the PubMed brick, which produces a ~ 66 GB Parquet table of all PubMed metadata. Thereafter, *any* researcher can obtain that fully processed dataset with a single biobricks install pubmed command, instead of each user individually spending hours or days to locate, download, and clean the raw data. Because each brick is version-controlled and cached, heavy transformations (e.g., converting files to Parquet, normalizing fields) are performed only once by the brick maintainer and then shared—avoiding repeated CPU cycles and bandwidth costs across the community. In one hands-on scenario we encountered, switching from a custom script to a pre-built brick reduced data ingestion time from “an afternoon of coding” to under 5 min (the remaining time being almost entirely network download)—a concrete illustration of how BioBricks can accelerate research while saving effort. BioBricks.ai also employs continuous integration tests to automatically run and verify each brick’s pipeline for quality control. This ensures that bricks remain reproducible and functional as their upstream sources or code dependencies change.

An apt analogy is to think of BioBricks as a package manager for data. Just as language-specific package managers (npm for JavaScript, pip for Python, CRAN for R, etc.) revolutionized software development by making it easy to share and reuse code libraries, BioBricks aims to streamline the sharing and reuse of data pipelines. Each brick is like a “data package” that can be installed on demand, bringing along metadata and dependency information. BioBricks.ai leverages open-source collaboration tools (GitHub for code hosting, issue tracking, version control) to encourage community-driven contributions and rapid iteration. Rather than competing with existing semantic web or integrative database efforts, BioBricks can be viewed as a complementary layer: it provides readily accessible, versioned raw datasets that could accelerate the building of semantic-web applications or new harmonized assets. In fact, BioBricks.ai has been used as part of the NSF Prototype Open Knowledge Network initiative demonstrating that bricks can feed into larger knowledge graph frameworks. Likewise, our ChemHarmony case study shows how one can create a new integrated asset (combining ~15 source databases) by chaining BioBricks dependencies, thus reducing redundant ETL effort when developing domain-specific knowledge graphs.

### 5.2 Limitations and future work

While BioBricks.ai offers significant advantages for public health data management, it faces several practical limitations. Here, we discuss these limitations and propose potential solutions for future development (see Table 3).

**Table 3:** Limitation and future improvements.

Limitation	Planned future improvements
Large datasets—some bricks produce very large outputs (multi-terabyte scale), which are impractical for users to download in full.	On-demand access: implement cloud-based query engines and data chunking so users can retrieve subsets of data without full downloads. Frequently accessed portions could be cached or summarized, and “lightweight” sample bricks could be offered for previewing gigantic datasets.
Data transfer costs—even with caching, repeatedly downloading big data files can incur high bandwidth costs for users or hosting providers.	Cost optimization: explore tiered storage/access solutions and collaborations with cloud providers to host popular bricks closer to users. We also plan to support distributed and mirror hosting of bricks to load-balance traffic and reduce single-point egress costs.
System complexity—new users face a learning curve in installing BioBricks.ai, setting up dependencies (DVC, Git, etc.), and understanding brick usage.	Improved onboarding: develop one-click remote development environments (e.g., cloud-based Jupyter or Docker images pre-loaded with BioBricks) so users can try bricks instantly without local setup. Enhanced documentation, tutorials, and community support (forums, chat) are also in progress to flatten the learning curve.
Data quality control—since bricks pull from diverse sources, ensuring consistent quality and format (and catching errors) across all bricks is challenging.	Automated QC and metadata: introduce automated data validation checks for each brick (e.g., schema conformance tests, basic sanity checks on values) to catch issues early. We are also formulating a standardized metadata schema (using LinkML, see below) for bricks to describe their contents, which will help in validating and comparing datasets. A community rating or review system for bricks could further incentivize high quality.
Real-time updates—keeping every brick up-to-date with changes in its upstream source can be labor-intensive, and lags may occur.	Auto-update & versioning: develop monitoring tools that detect when source data has changed (new releases, etc.) and trigger automated pipeline runs. Improved versioning practices will be implemented so that users can be notified of important updates or breaking changes in bricks.
Usage tracking—currently, maintainers have limited insight into which bricks are used most or how they are being utilized. Such feedback could guide development priorities.	Telemetry (Opt-In): add an opt-in usage logging feature within the BioBricks client that can report anonymous statistics (e.g., which bricks were installed how often). This can help identify highly valuable bricks and also provide credit to contributors, while strictly respecting user privacy.
Integration with workflows—users may find it non-trivial to integrate BioBricks data retrieval into their existing analysis pipelines or tools.	APIs and connectors: provide language-specific SDKs/APIs (for Python, R, etc.) to allow programmatic access to bricks in notebooks and scripts. Develop plug-ins or adapters for popular bioinformatics platforms (for example, integrating BioBricks with Galaxy workflows or RStudio projects). We will also seek compatibility with emerging data standards and formats to ease integration.
Scalability—as the number of bricks and users grows, performance bottlenecks could arise (whether in the central index, package registry, or data hosting).	Scalable architecture: invest in a more distributed architecture for the backend, including load-balanced servers and perhaps decentralized data storage (P2P or cloud CDN). Performance profiling and optimization of the client and DVC pipelines is ongoing to ensure the system scales to hundreds of bricks and large user bases.

## 6 Conclusion

BioBricks.ai represents a significant advancement in the management and distribution of biomedical research and public health data, offering a solution to the longstanding challenges of data fragmentation, accessibility, and reproducibility in the life sciences. By extending the principles of part reuse and standardization to public health data management, BioBricks.ai is poised to accelerate scientific discovery and innovation across various fields, including drug discovery, toxicology, biochemistry, and public health research.

## Author’s note

*CLI Package*: BioBricks.ai command line interface is on PyPI pipx install biobricks.

*Client Packages*: The R and python packages can be installed from cran and PyPI.

*Source Code*: The BioBricks.ai command line interface is github.com/biobricks-ai/biobricks.

*Operating System*: Biobricks.ai supports Windows, Mac, and Linux.

*Usage Restrictions*: BioBricks.ai is open source with an MIT license.

Please read more detailed used cases on https://insilica.co/posts/.

## Data Availability

The original contributions presented in the study are included in the article/Supplementary material, further inquiries can be directed to the corresponding author.

## Footnotes

^1^ https://dvc.org/doc/use-cases/data-registry

^2^ https://github.com/biobricks-ai/brick-template

^3^ https://github.com/biobricks-ai/hgnc

^4^ https://github.com/biobricks-ai/clinvar
