# Supplementary material for: BioBricks.ai: a versioned data registry for life sciences data assets
Source: Front Artif Intell. 2025 Aug 13;8:1599412. doi: 10.3389/frai.2025.1599412 (PMC12380851; doi:10.3389/frai.2025.1599412)
Supplement: Supplementary file 1 [file Data_Sheet_1.PDF]

Appendix A: User Guide – Installing, Using, and Developing with BioBricks.ai

This appendix provides practical instructions for users and developers to install BioBricks.ai, access data bricks, and build new bricks. It is intended for readers interested in applying BioBricks.ai in their own data workflows.

A.1 Installation and Setup

BioBricks.ai is distributed as a Python package and is compatible with macOS, Linux, and Windows. We recommend using pipx to isolate the installation from your global Python environment.

Steps to Install and Configure BioBricks.ai:

```bash
pipx install biobricks         # install CLI in isolated environment
biobricks configure            # create and configure your brick library
biobricks install <brickname>  # install a specific brick, e.g. hgnc
```

You may also use pip install biobricks if pipx is unavailable. During configuration, you will be prompted to create a library path and enter your API token (available at https://biobricks.ai). Data will be stored locally in your configured library path with a cache subdirectory for efficient reuse across bricks.

A.2 Using Installed Bricks

After installation, each brick provides access to one or more data assets (e.g., Parquet tables, SQLite databases). Assets can be listed and accessed using the CLI or programmatically.

Example: Accessing HGNC Brick in Python

```python
import biobricks as bb
import pandas as pd
```

```
hgnc = bb.assets('hgnc')  # Load assets from the HGNC brick
df = pd.read_parquet(hgnc.hgnc_complete_set_parquet)
print(df.head())
```

Each asset is exposed as a named attribute (e.g., hgnc_complete_set_parquet) within the brick's namespace. Supported data formats include .parquet, .sqlite, and .hdt.

```
        hgnc_id    symbol                                        name         locus_group  ... gtrnadb         agr                       mane_select      gencc
0        HGNC:5      A1BG                        alpha-1-B glycoprotein  protein-coding gene  ...    None      HGNC:5   ENST00000263100.8|NM_130786.4       None
1    HGNC:37133  A1BG-AS1                          A1BG antisense RNA 1       non-coding RNA  ...    None  HGNC:37133                              None       None
2    HGNC:24086      A1CF              APOBEC1 complementation factor  protein-coding gene  ...    None  HGNC:24086   ENST00000373997.8|NM_014576.4       None
3        HGNC:7       A2M                        alpha-2-macroglobulin  protein-coding gene  ...    None      HGNC:7  ENST00000318602.12|NM_000014.6     HGNC:7
4    HGNC:27057   A2M-AS1                           A2M antisense RNA 1       non-coding RNA  ...    None  HGNC:27057                              None       None
...         ...       ...                                          ...                  ...  ...     ...         ...                               ...        ...
43713 HGNC:25820    ZYG11B   zyg-11 family member B, cell cycle regulator  protein-coding gene  ...    None  HGNC:25820   ENST00000294353.7|NM_024646.3 HGNC:25820
43714 HGNC:13200       ZYX                                        zyxin  protein-coding gene  ...    None  HGNC:13200  ENST00000322764.10|NM_003461.5       None
43715 HGNC:51695    ZYXP1                            zyxin pseudogene 1           pseudogene  ...    None  HGNC:51695                              None       None
43716 HGNC:29027    ZZEF1  zinc finger ZZ-type and EF-hand domain contain...  protein-coding gene  ...    None  HGNC:29027   ENST00000381638.7|NM_015113.4       None
43717 HGNC:24523     ZZZ3              zinc finger ZZ-type containing 3  protein-coding gene  ...    None  HGNC:24523   ENST00000370801.8|NM_015534.6       None

[43718 rows x 54 columns]
```

Figure A. 1 Data in hgnc.hgnc_complete_set_parquet

A.3 Brick Repository Structure

Each BioBrick is implemented as a Git repository. The standard structure includes:

```bash
/brick              # Final data outputs (e.g., .parquet, .sqlite)
.dvc/               # DVC tracking files
.bb/                # Metadata and dependencies
dvc.yaml            # Defines build stages (ETL pipeline)
dvc.lock            # Records hashes of input/output files
```

Assets are built via DVC pipelines to ensure reproducibility. Dependencies between bricks are declared in .bb/dependencies.txt.

A.4 Example: Building the SMRT Brick

To build a brick locally, clone a brick repository (e.g., SMRT), and run the ETL pipeline:

bash

```
git clone https://github.com/biobricks-ai/SMRT.git
cd SMRT
dvc repro   # Runs ETL pipeline: check → download → process
```

Pipeline stages:

1. **Status** – Checks for upstream data changes
2. **Download** – Fetches raw data
3. **Process** – Transforms data into the final brick format

Bricks can also be built or explored in cloud environments (e.g., GitHub Codespaces).

A.5 Publishing New Bricks (For Developers)

Developers invited to contribute bricks can use the brick-template repository to scaffold a new dataset.

Typical process:

1. Clone the template
2. Customize the ETL stages in `dvc.yaml`
3. Run the pipeline `dvc repro`
4. Push built assets: `dvc push -r s3.biobricks.ai`

Submit the repository for review by the BioBricks core team. A new `biobricks push` feature (coming soon) will support broader community submissions.

A.6 Resources

Website: https://biobricks.ai
CLI Package (Python): https://pypi.org/project/biobricks/
R Client: Available via CRAN (`install.packages("biobricks")`)

Documentation: https://docs.biobricks.ai
GitHub Repos: https://github.com/biobricks-ai
License: MIT (Open Source)
For further questions, refer to the documentation or contact the BioBricks.ai team via GitHub or the support channels listed on the website.
